# Metabolic profiling of cytotoxic metabolites from five Tabebuia species supported by molecular correlation analysis

**DOI:** 10.1038/s41598-021-87695-w

**Published:** 2021-04-16

**Authors:** Seham S. El-Hawary, Rabab Mohammed, Ahmed F. Tawfike, Sameh Fekry AbouZid, Marwa A. Taher, Usama Ramadan Abdelmohsen, Elham Amin

**Affiliations:** 1 Department of Pharmacognosy, Faculty of Pharmacy, Cairo University, Cairo, Egypt; 2 Department of Pharmacognosy, Faculty of Pharmacy, Beni-Suef University, Beni-Suef, 62514 Egypt; 3 Department of Pharmacognosy, Faculty of Pharmacy, Helwan University, Cairo, 11795 Egypt; 4 Department of Computational and Analytical Science, Molecular Discovery Group, Rothamsted Research, Harpenden, AL5 2JQ England, UK; 5 Department of Pharmacognosy, Faculty of Pharmacy, Heliopolis University, Cairo, 11785 Egypt; 6 Department of Pharmacognosy, Faculty of Pharmacy, Nahda University, Beni-suef, Egypt; 7 Department of Pharmacognosy, Faculty of Pharmacy, Minia University, Minya, Egypt; 8 Department of Pharmacognosy, Faculty of Pharmacy, Deraya University, Universities Zone, New Minia City, Minia Egypt; 9 Department of Medicinal Chemistry and Pharmacognosy, College of Pharmacy, Qassim University, Buraidah, 52471 Saudi Arabia

**Keywords:** Chemical biology, Plant sciences

## Abstract

*Tabebuia* is the largest genus among the family Bignoniaceae. *Tabebuia* species are known for their high ornamental and curative value. Here, the cytotoxic potential of extracts from the leaves and stems of five *Tabebuia* species was analyzed. The highest activity was observed for *T. rosea* (Bertol.) DC*.* stem extract against HepG2 cell line (IC_50_ 4.7 µg/mL), *T. pallida* L*.* stem extract against MCF-7 cell line (IC_50_ 6.3 µg/mL), and *T. pulcherrima* stem extract against CACO2 cell line (IC_50_ 2.6 µg/mL). Metabolic profiling of the ten extracts using liquid chromatography–high-resolution mass spectrometry for dereplication purposes led to annotation of forty compounds belonging to different chemical classes. Among the annotated compounds, irridoids represent the major class. Principle component analysis (PCA) was applied to test the similarity and variability among the tested species and the score plot showed similar chemical profiling between the leaves and stems of both *T. pulcherrima* and *T. pallida* L. and unique chemical profiling among *T. rosea* (Bertol.) DC., *T. argentea* Britton, and *T. guayacan* (Seem.) Hemsl*.* leaf extracts and the stem extract of *T. rosea* (Bertol.) DC. Additionally, a molecular correlation analysis was used to annotate the bioactive cytotoxic metabolites in the extracts and correlate between their chemical and biological profiles.

## Introduction

Bignoniaceae, the trumpet vine or trumpet creeper family, is a large, widely distributed family named after the genus *Bignonia* relative to Jean-Paul Bignon^1^. Bignoniaceae contains about 110 genera, the most famous of which are *Tecoma, Catalpa, Tabebuia,* and *Jacaranda,* and 650 species, most of which are ornamentals and contain a wide variety of constituents with diverse pharmacological activities^2,3^. The genus *Tabebuia,* the largest Bignoniaceae genus, contains about 100 species and is known by rural populations to have therapeutic activity^4^. Most of its species are used as anti-inflammatory, anticancer, and antimicrobial agents in rural areas of Colombia, Bolivia, Brazil, and other Latin American countries^5^. “Taheebo” or “lapacho”, the herbal product of *Tabebuia* bark, is traditionally used to treat ulcers, syphilis, gastrointestinal problems, candidiasis, diabetes, prostatitis, constipation, allergies^6^ and also has anti-cancerous properties^7^.

The main constituents of *Tabebuia* spp. bark extracts are naphthoquinones, furanonaphthoquinones, anthraquinones, benzoic acid derivatives, benzaldehyde derivatives, iridoids, coumarins, and flavonoids^8^. Remarkably, the β-lapachone, a naphthoquinone found in most *Tabebuia,* species, is now in the clinical trial and drug development phase as a plant-derived anticancer agent^9^. Natural products are important source of inspiration for discovering anticancer candidates. About 60% of drugs used nowadays to treat cancer were isolated from natural products, and more than 3000 plants have been reported to have anticancer activity^10^.

However, natural extracts require complicated process to obtain pure identified natural product and liquid chromatography–mass spectrometry is powerful analytical tool for assist the study of plant extracts; it detects a wide range of chemical compounds simultaneously without the need for a tedious isolation procedure^11^. In addition, dereplication process, perform a rapid annotation of known secondary metabolites and their quantification in crude extracts using database searching, to screen samples, which saves time and reduces redundancy during natural product discovery programs^12^. Metabolic profiling is applied to annotate and biotechnologically optimize the production of pharmacologically active secondary metabolites^13^, using multivariate data analysis like Principle component analysis (PCA) to reduce dimensionality of the data and highlights trends, groups or clusters^14^ along with molecular correlation analysis to pinpoint the putatively active components.

Consequently, the present work, illustrate the cytotoxic potential of the leaves and stems of five *Tabebuia* species against three different cell lines [HepG2 (human hepatoma), MCF-7 (human breast adenocarcinoma), and CACO2 (human colon adenocarcinoma) cells]. Metabolic profiling and dereplication approaches were used in order to explore the differences in secondary metabolite patterns between the ten tested samples of *Tabebuia* species and to develop a database of annotated compounds with a chemical survey of compounds responsible for cytotoxic activity.

## Results and discussion

### Cytotoxic activity of plant extracts

The results of the cytotoxic activity of the ten plant samples against the three cancer cell lines, HepG2, MCF-7, and CACO2, are represented in (Supplementary Table S1). All extracts except *T. guayacan* (Seem.) Hemsl. stem extract exhibited significant cytotoxic activity against the HepG2 cell line at the examined concentrations, with *T. rosea* (Bertol.) DC. stem extract being the most potent (IC_50_ 4.7 µg/mL). In the MCF-7 cell line, all tested plant samples displayed significant cytotoxic activity except *T. guayacan* (Seem.) Hemsl. leaf extract and *T. rosea* (Bertol.) DC. leaf and stem extracts, with *T. pallida* L. stem extract being the most active (IC_50_ 6.3 µg/mL). All tested extracts displayed potent activity against the CACO2 cell line, but *T. pulcherrima* stem extract was the most potent (IC_50_ 2.6 µg/mL).

Surveying the relevant literature, it was found that the traditional use of *Tabebuia* species for treating cancer began in Brazil in the 1960s and led to increased sales of the bark and wood of the trunk of *T. impetiginosa* (Mart. Ex DC.) Standl., *T. rosea* (Bertol.) DC., and *T. serratifolia* (Vahl)^15^. The in vitro antitumor potential of the total alkaloid extract of *T. rosea* (Bertol.) DC*.* leaves was evaluated in human leukemic cells (MOLT-4)^16^ and the anticancer properties of *T. pallida* L. leaves were evaluated in Ehrlich ascites carcinoma^17^. In terms of bioactive compounds, naphthoquinones, particularly β-lapachone, have been reported to selectively induce apoptotic cell death in various cancers, including breast cancer, prostate cancer, and leukemia^18^. According to the United States National Cancer Institute plant screening program, crude extract is reported to have in vitro cytotoxic activity if the IC_50_ is < 30–40 μg/mL^19^. All of the ten extracts tested in the present study showed potent in vitro cytotoxic activity, except *T. guayacan* (Seem.) Hemsl. leaf extract and *T. rosea* (Bertol.) DC. leaf and stem extracts, which showed no cytotoxic activity against MCF-7 cells, and *T. guayacan* (Seem.) Hemsl. stem extract, which showed no cytotoxicity against HepG2 cells.

### LC–HRESIMS

**Figure 1:**
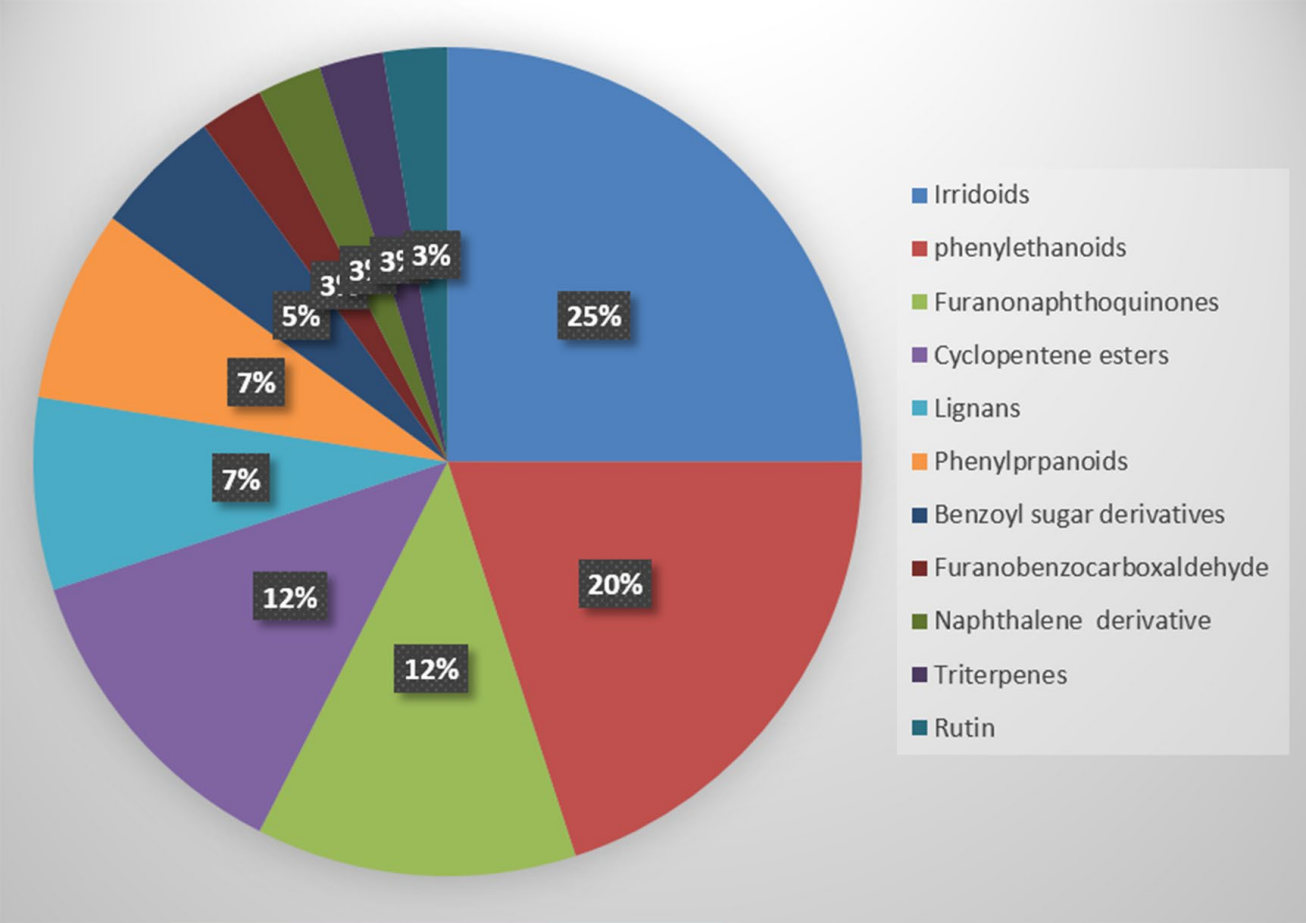
Percentage of different classes of metabolites annotated from *Tabebuia* species.

The metabolic profiling of the five *Tabebuia* species against the Dictionary of Natural Products (DNP) and METLIN databases resulted in annotation of forty compounds belonging to different chemical classes (Fig. 1). Iridoids and phenylethanoids were the major detected chemical classes. “The entire list of 40 compounds can be found as Supplementary Table S2 and Fig. S2 online, the presence of these compounds among the different species under investigation are also available in Supplementary Table S3 online”.

“For our quality check, as an alternative to an internal standard to monitor the alignment of peaks, we used rutin (C_27_H_30_O_16_, m/z 609.1459 [M−H]-, tR 10.65), which is an universal endogenous plant metabolite, and which belongs to more than 30 plant families. Moreover, we have reported the presence of rutin from *Tabebuia aurea* in our previous work^20^. The figure (S1) shows the retention time alignment of rutin’s peak in all samples, which was within the acceptable RT tolerance threshold according to our previous work^21^. It is of note, however, that while the use of rutin allowed us to monitor for RT variability, it did not allow for the correction of potential analytical errors of injection and sample preparation”.

### Metabolic profiling and molecular correlation analysis

**Figure 2:**
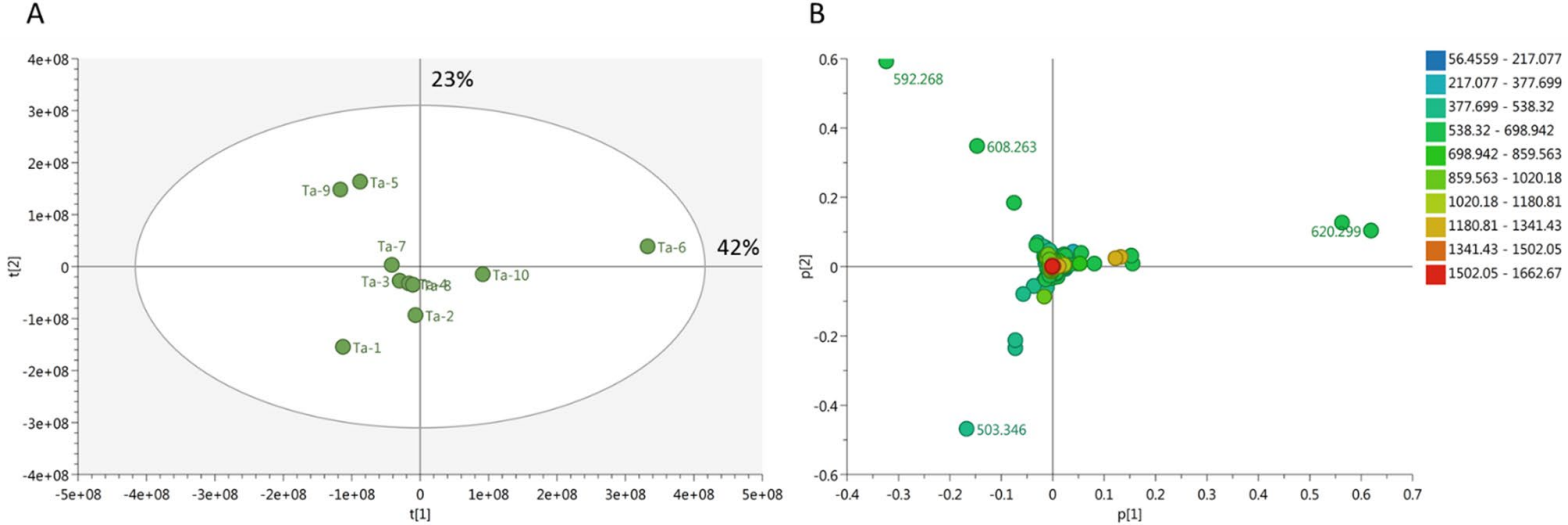
Principal component analysis (PCA) (Cytoscape software, version 3.4.0) score plot showed a respective total variance of 42% and 23% for PC1 and PC2, repectively. (**A**) PCA score plot of crude *Tabebuia* extracts; (**B**) PCA loading plot showing discriminatory metabolites.

To achieve the best coverage of the *Tabebuia* metabolome, the positive and negative mass spectral data were merged into one data matrix. The data were processed using MZmine2 according to a method developed previously in our lab^22^ and transferred to an in-house database Excel file with a built-in DNP database for dereplication purposes. PCA was applied to the data to test the similarity and/or variation of the chemical profiles of the tested species. PCA is an unsupervised multivariate data analysis that aims to reduce the dimensionality of data to reveal clusters, groups, and/or outliers among observations^23^. The PCA score plot (Fig. 2A) showed a respective total variance of 42% and 23% for PC1 and PC2 and demonstrated clustering of *T. pulcherrima* and *T. pallida* L. leaf and stem extracts (Ta-3, Ta-4, Ta-7, and Ta-8, respectively). This indicates that these extracts have similar chemical profiles. Further, PCA showed a dispersal of *T. rosea* (Bertol.) DC. (Ta-5), *T. argentea* Britton (Ta-9), and *T. guayacan* (Seem.) Hemsl. (Ta-1) leaf extracts as well as the stem extract of *T. rosea* (Bertol.) DC. (Ta-6), which indicates that these extracts differ in terms of their chemical profiles. The PCA loading plot (Fig. 2B) highlights the fact that metabolites contribute to this variation, as the *T. rosea* (Bertol.) DC*.* (Ta-5) and *T. argentea* Britton (Ta-9) leaf extracts were characterized by xanthone-like molecules corresponding to C_34_H_40_O_9_, which is equivalent to, e.g., moreollic acid or scortechinone A at m/z (retention time in minutes) 592.268 [M^+^] (t_R_ 29.10) and 608.263 [M^+^] (t_R_ 28.84) for C_34_H_40_O_10,_ equivalent to scortechinone M. Moreollic acid from *Garcinia hanburyi* was previously reported to show cytotoxicity against cervical cancer cell lines (HeLa)^24^. Scortechinone A and M were also identified previously from *G. scortechinii*^15^. In addition, the stem extract of *T. rosea* (Bertol.) DC*.* (Ta-6) was characterized by a nimbolinin-type limonoid at m/z 620.299 [M^+^] (t_R_ 23.49) corresponding to C_36_H_44_O_9_, which was reported from *Melia azedarach* and has been shown to exhibit cytotoxicity against the leukemia cell line HL-60^25^. *T. guayacan* (Seem.) Hemsl*.* (Ta-1) leaf extract was characterized by a molecule at m/z 503.346 [M^+^] (t_R_ 23.41) corresponding to C_26_H_49_NO_8._ The only hit for this molecule in the DNP database was the fungal metabolite thermolide D, which was filtered out due to taxonomic irrelevance.

**Figure 3:**
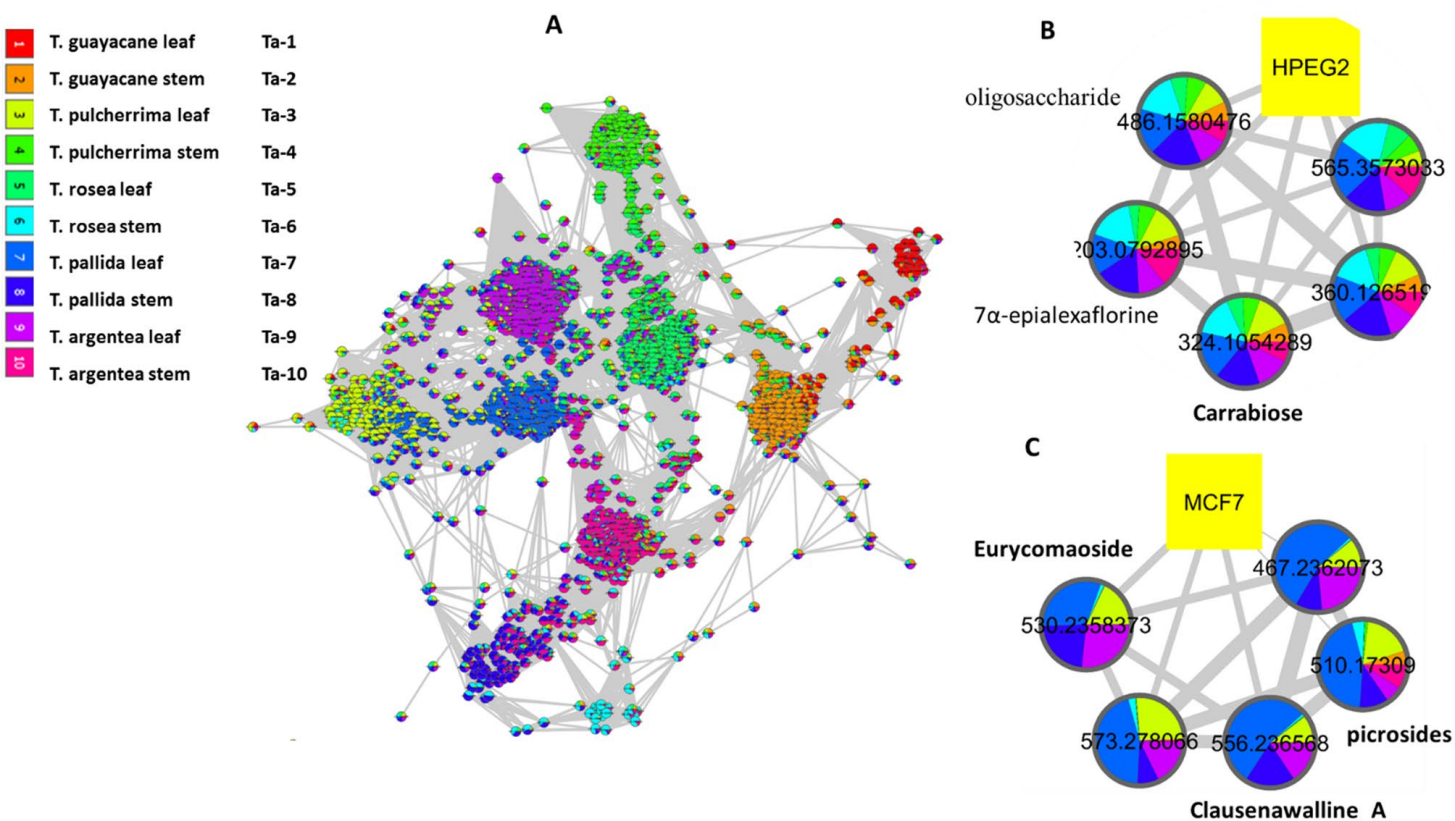
Molecular correlation analysis (Cytoscape software, version 3.4.0). (**A**) Whole molecular correlation analysis showing molecular interactions/grouping; (**B**) extracted analysis showing molecules highly correlated with HepG2 cytotoxicity; (**C**) extracted analysis showing molecules highly correlated with MCF-7 cytotoxicity. Nodes are labeled with the molecular weights of the compounds that they represent. The width of the edges connecting nodes is proportional to the strength of interactions.

The dereplication study of the discriminatory molecules was found to be matching with the biological investigation results, as *T. rosea* (Bertol.) DC. (Ta-5), *T. argentea* Britton (Ta-9) leaf extracts as well as the stem extract of *T. rosea* (Bertol.) DC. (Ta-6) were found cytotoxic to one or more of the tested cancer cell lines i.e. HepG2, MCF-7 and CACO2.The biological investigation of *Tabebuia* species showed that *T. rosea* (Bertol.) DC. stem (Ta-6) is the most cytotoxic to HepG2 cancer cell line followed by *T. pallida* L. leaf and *T. argentea* Britton stem (Ta-7 and Ta-10, respectively). Whereas, Ta-8 and Ta-3 (*T. pallida* L. stem and *T. pulcherrima* leaf) were the most active against MCF-7 cancer cell line. Furthermore, *T. pulcherrima* stem (Ta-4) was the most cytotoxic to CACO2. The biological data refer that *T. pallida* L. leaf (Ta-7) might possess the most cytotoxic agents followed by its stem extract (Ta-8), *T. rosea* (Bertol.) DC. stem (Ta-6) and *T. pulcherrima* leaf and stem (Ta-3 and Ta-4). To annotate the agents responsible for this cytotoxicity, the spectral data, integrated with the metadata (biological data), were analyzed via a similarity correlation analysis^26^. The molecular correlation analysis used a Pearson correlation coefficient to detect molecules linked with observed cytotoxicity. In the analysis in Fig. 3A, metabolites that correlate with each other and with the biological data are labeled with their molecular weights and colored in the pie chart according to the concentration of the metabolite in each of the tested species. The Pearson correlation coefficient was set to 0.8. An extracted analysis (Fig. 3B) shows that the molecules highly correlated with HepG2 cytotoxicity at m/z [M^+^] (retention time in minutes) 565.357 (t_R_ 26.55), 324.105 (t_R_ 1.75), 360.127 (t_R_ 1.74), 486.158 (t_R_ 1.75), and 203.079 (t_R_ 1.73). The dereplication studies revealed that the compound at m/z 324.105 corresponds to C_12_H_20_O_10_, equivalent to the disaccharide Carrabiose, which was previously reported to show antitumor activity in murine mammary adenocarcinoma^27^. Meanwhile, the compound at m/z 486.158 corresponds to C_18_H_30_O_15_, equivalent to an oligosaccharide molecule, that was found to be responsible for the inhibition of melanogenesis in murine B16 melanoma cells^28^. In addition, the compound at m/z 203.079 corresponds to C_8_H_13_NO_5_, equivalent to 7α-epialexaflorine from leaves of *Alexa grandiflora,* reported to have antifungal properties. The remaining metabolites, i.e., those at m/z 565.357 and 360.127, have not previously been annotated, indicating that they are new chemical entities and that further study may be necessary to elucidate their structures. In terms of MCF-7 cytotoxicity, the extracted analysis (Fig. 3C) showed the active agents involved. The molecules at m/z [M^+^] (retention time in minutes) 510.173 (11.25), 530.236 (10.37), 556.237 (9.18), 573.278 (12.53), and 467.236 (9.52) were all found to be linked to MCF-7 cytotoxicity. These metabolites were annotated as follows: the compound at m/z 510.173 corresponds to C_24_H_30_O_12_, equivalent to iridoid glycosides, i.e., picrosides, which have been reported to inhibit the invasion and migration of MCF-7 breast cancer cells^29^. The compound at m/z 530.236 corresponds to C_25_H_38_O_12_, equivalent to a quassinoid-type glycoside, eurycomaoside^30^. That at m/z 556.237 corresponds to C_36_H_32_N_2_O_4_, equivalent to the carbazole alkaloid clausenawalline A, which was reported to show cytotoxicity in oral cavity cancer (KB), breast cancer (MCF-7), and small cell lung cancer (NCI-H187)^31^. Finally, the compounds at m/z 573.278 and at m/z 467.236 were not annotated previously.

**Figure 4:**
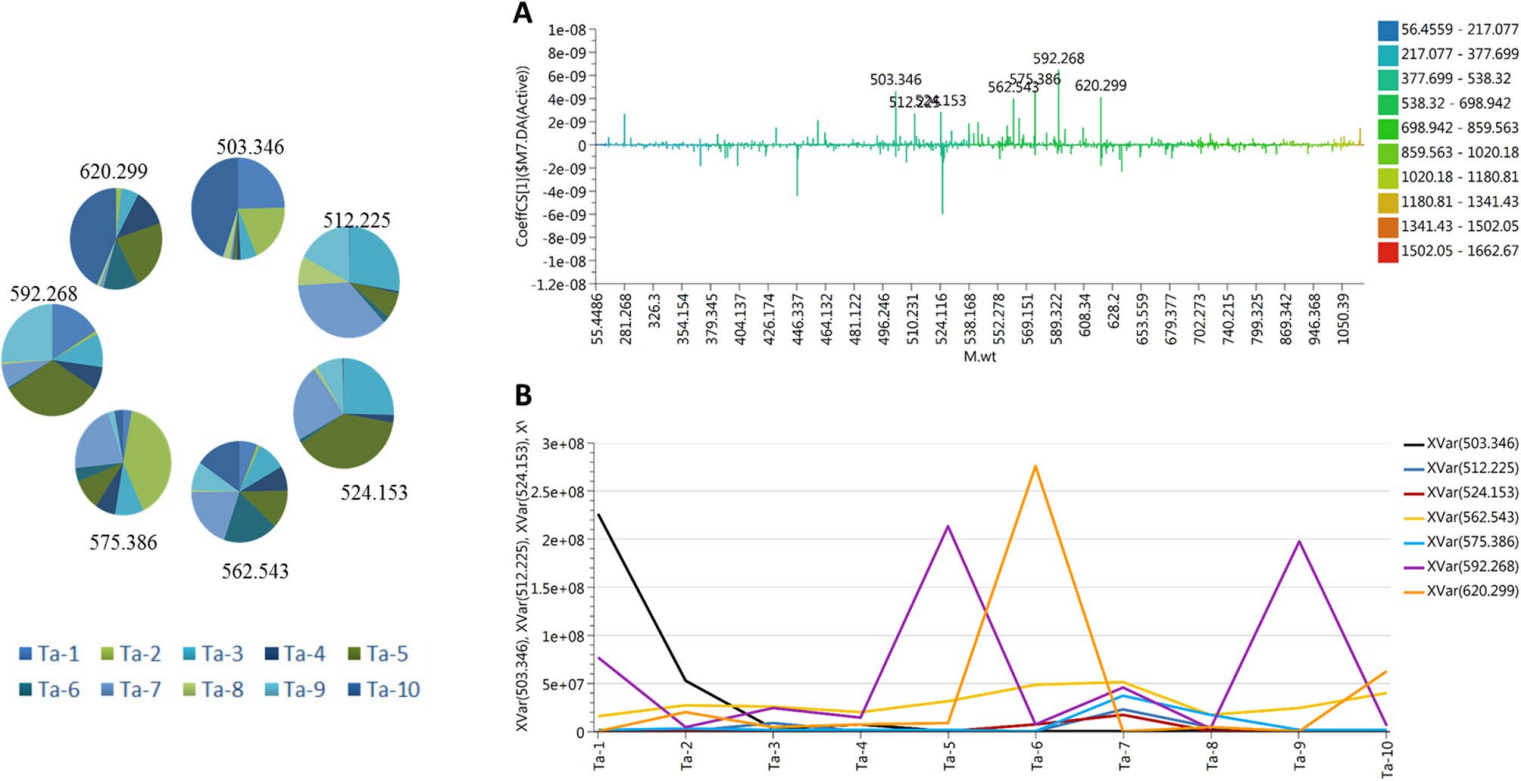
(**A**) Coefficient of variation of metabolites linked to CACO2 cytotoxicity; (**B**) intensity of the top correlated metabolites across the tested species (Cytoscape software, version 3.4.0).

Unfortunately, relationships between the spectral data and CACO2 metadata were not detected using the similarity correlation analysis. This might indicate that there is no one specific molecule or class of molecules that mediate the biological activity in this case, but a synergism of more than one molecule and/or class of compounds. However, in order to check which metabolites may contribute to cytotoxicity against CACO2 cells, an orthogonal partial least square discrimination analysis module was created. The model has a strong goodness of fit R^2^ = 0.99 and a goodness of prediction Q^2^ = 0.91. The coefficient of variation plot (Fig. 4) is a very useful tool for comparing the magnitude of a variable to its reliability, where regression coefficients related to scaled and centered X-variables are displayed. This scaling of the data makes the coefficients comparable. Thus, these coefficients express how strongly Y is correlated to the systematic part of each of the X-variables. The molecules highly correlated with CACO2 cytotoxicity were checked and only those with high coefficients of variation and 95% confidence level limits not crossing zero were chosen. The metabolites highly correlated with CACO2 cytotoxicity were at m/z [M^+^] (retention time in minutes) 503.346 (23.41), 512.225 (11.84), 524.153 (10.35), 562.543 (29.15), 575.386 (23.92), 592.268 (29.11), and 620.299 (27.21). The dereplication study revealed that the compounds at m/z 512.225 and 524.153 correspond to C_25_H_36_O_11_ and C_24_H_28_O_13_, respectively, which are equivalent to the iridoid glycosides 10-*O*-foliamenthoylaucubin and nudifloside, respectively. These compounds were previously reported to have antitumor and antimicrobial properties^32,33^. Some molecules were detected previously by PCA analysis, i.e., those at m/z 592.268 and 620.299, which correspond to xanthone and limonoid, respectively. The rest of the detected molecules have not previously been annotated, and further study will be necessary to elucidate their structures. Figure 4B illustrates the intensity of the significant metabolites across *Tabebuia* species; those at m/z 503.346, 592.268, and 620.299 were the most intense among all of the highly correlated metabolites. In summary, metabolic study is a powerful tool that rapidly annotated the bioactive metabolites that may mediate in the demonstrated cytotoxicity of *Tabebuia* extracts against MCF-7, HepG2, and CACO2 cancer cell lines. The dereplication study relied on precise molecular formula prediction and chemotaxonomic filtration to minimize the number of hits per molecular formula, leading to the tentative annotation of the top hits^34^. Searching the literature for the bioactivities reported previously for these molecules proved they could be mediators of the exhibited cytotoxicity.

## Material and methods

### Collection of plant samples

The leaves and stems of five *Tabebuia* species were collected by taking permission from the directors of two different botanical gardens: *T. argentea* Britton and *T. guayacan* (Seem.) Hemsl. were collected from Al-Zohriya garden, Zamalek, Cairo Governorate, Egypt; and *T. pulcherrima, T. pallida* L., and *T. rosea* (Bertol.) DC. were collected from the botanical garden in Aswan (El-Nabatate island), Aswan, Egypt. All plant samples were identified by Prof. Dr. Abdel-Halim Mohammed (Professor of Agriculture, Flora Department, Agricultural Museum, Dokki, Giza, Egypt) and collected according to plant collections guidelines of Alberta Native Plant Council 2006. Voucher specimen kept in the Botanical garden in Aswan, Aswan, Egypt with number (Ta 1–10) were deposited. Plant materials were washed separately with fresh water and dried in the shade with occasional sun for several days. The dried materials were ground into coarse powder by a grinding machine and the materials were stored at room temperature for future use.

### Preparation of extracts for metabolites dereplication and cytotoxic activity analysis

About 300 g of the powdered material of each plant was macerated separately in 500 mL of 70% ethanol in sealed amber-colored extraction bottles. The bottles were kept for 7 days with occasional shaking and stirring. Then, the extracts were separately filtered through a fresh cotton plug. The filtrates were concentrated using a rotary evaporator under reduced pressure at 45 °C.

### Cytotoxic activity testing

The cytotoxicity of the plant sample extracts was evaluated in cell lines using a 3-(4,5-dimethylthiazol-2-yl)-2,5-diphenyltetrazolium bromide (MTT) assay^35^. HepG2 (human hepatoma), MCF-7 (human breast adenocarcinoma), and CACO2 (human colon adenocarcinoma) cells were maintained in RPMI medium (Merck, Darmstadt, Germany) supplemented with 10% fetal bovine serum. MCF-7 cells were cultured at 37 °C and 5% (v/v) CO_2_ in RPMI 1640 medium supplemented with 5% (v/v) fetal bovine serum, 1% (w/v) l-glutamine, 1% sodium pyruvate, and 0.4% (w/v) antibiotics (50 U/mL penicillin, 50 mg/mL streptomycin). Cells were obtained from the American Type Culture Collection (Rockville, MD, USA; HPACC, Salisbury, UK) and sub-cultured twice per week. All chemicals and reagents were purchased from Sigma Aldrich (Darmstadt, Germany). To normalize cell viability values, each plate included a triplicate of cells treated with the compound carrier dimethyl sulfoxide to define 100% viability as well as a triplicate of cells incubated with a cytotoxic mixture [200 ng/mL tumor necrosis factor, 200 ng/mL CD95L (Fas ligand), 200 ng/mL tumor necrosis factor–related apoptosis-inducing ligand, 25 g/mL cycloheximide, 1% (w/v) sodium azide] to define maximal cell death and thus 0% viability. All other viability values were normalized according to the averages of these triplicates and analyzed using Graph Pad Prism 5 software (La Jolla, CA, USA). 5-Flurouracil was used as a positive control.

### Liquid chromatography–high-resolution electrospray ionization mass spectrometry

One mg of each crude extract (of the ten samples under investigation) was weighted using sensitive electric balance (Sartorius, type 1712, Germany) and dissolved in 1 mL HPLC grade methanol then it was analyzed according to Abdelmohsen et al.^36^ on an Acquity Ultra Performance Liquid Chromatography system coupled to a Synapt G2 HDMS quadrupole time-of-flight hybrid mass spectrometer (Waters, Milford, MA, USA). Chromatographic separation was performed on a BEH C18 column (2.1 × 100 mm, 1.7 µm particle size; Waters, Milford, MA, USA) with a guard column (2.1 × 5 mm, 1.7 µm particle size) and a linear binary solvent gradient of 0–100% eluent B over 6 min at a flow rate of 0.3 mL min^–1^ using 0.1% formic acid in water (v/v) as solvent A and acetonitrile as solvent B. All reagents were of analytical grade and were purchased (Fisher Scientific, Hemel Hempstead, UK). The injection volume was 2 µL and the column temperature was 40 °C. After chromatographic separation, the metabolites were detected by mass spectrometry using electrospray ionization in the positive mode; the source operated at 120 °C. The electrospray ionization capillary voltage was set to 0.8 kV, the sampling cone voltage was set to 25 V, and nitrogen was used as the desolvation gas (at 350 °C and a flow rate of 800 L h^–1^) and the cone gas (at a flow rate of 30 L h^–1^). The mass range for time-of-flight mass spectrometry was set to m/z (mass-to-charge ratio) 50–1200. Ms converter software was used in order to convert the raw data into divided positive and negative ionization files. Obtained files were then subjected to the data mining software MZmine 2.12 (https://bmcbioinformatics.biomedcentral.com/articles/10.1186/1471-2105-11-395) for deconvolution, peak picking, alignment, deisotoping, and formula prediction. For the combination of negative and positive ionization mode data files that were generated by MZmine, Excel macros were used. Both negative and positive ionization switch modes were used to include the highest number of metabolites from the investigated methanol extracts subjected to LC–HR-ESIMS analysis. The dereplication was achieved for each m/z ion peak with metabolites recorded in the customized databases based on established parameters (m/z threshold of ± 3 ppm and retention time), consequently, the number of the remaining unknown metabolites in each species was refine. The raw data was processed, aligned and merged into one dataset according to the method previously developed in our lab^20,22,37^.

The molecular correlation analysis was created via specific application of the cytoscape software (version 3.4.0) as reported in our previous work^25^. The Expression Correlation app, implemented by Sander Group (Computational Biology Center, Memorial Sloan-Kettering Cancer Center, New York City), was used to compute a similarity network from either observation (active fractions) or their corresponding features (m/z) in data matrix. Similarity network is using the Pearson correlation coefficient to link the active fractions (observations correlation network) or their corresponding metabolites (features correlation network). A feature correlation network was created to explore which of the metabolites will be highly correlated with the bioactivity (represented by percentage of viability). The negative correlation threshold was set to 0.7 whereas the positive one was neglected. The network was mapped via organic y files layout, a kind of spring-embedded algorithm.

## Conclusion

The metabolic profiling of five *Tabebuia* species, using LC-HRMS lead to annotation of 40 compounds belonging to ten different chemical classes. PCA was effectively employed to test the similarity and/or variation of the chemical profiles of the tested species. PCA results demonstrated similar chemical profiles of *T. pulcherrima* and *T. pallida* L., and a unique chemical profile of *T. argentea* Britton, *T. guayacan* (Seem.) Hemsl., and *T. rosea* (Bertol.) DC. The biological investigation results, indicated *T. rosea* (Bertol.) DC. stem extract as the most cytotoxic to HepG2 cancer cell line, whereas, *T. pallida* L. stem was the most active against MCF-7 cancer cell line. Furthermore, *T. pulcherrima* stem extract was the most cytotoxic to CACO2. The molecular correlation analysis highlighted the compounds responsible for cytotoxic activity against HepG2, MCF-7 and CACO2 cell lines. Interestingly, the molecules detected at m/z 565.357 and 360.127, directly linked to HepG2 activity and that detected at m/z 573.278 directly linked to MCF-7 activity, were not previously reported, suggesting that a new chemical structure still to be discovered.

## Supplementary Information

Supplementary Information 1.

Supplementary Information 2.
