# Supplementary material for: Metabolic profiling of cytotoxic metabolites from five Tabebuia species supported by molecular correlation analysis
Source: Sci Rep. 2021 Apr 16;11:8405. doi: 10.1038/s41598-021-87695-w (PMC8052319; doi:10.1038/s41598-021-87695-w)
Supplement: Supplementary file 2 — Supplementary Information 2. [file 41598_2021_87695_MOESM2_ESM.docx]

**Supplementary Data**

***Metabolic profiling of cytotoxic metabolites from five Tabebuia species supported by molecular correlation analysis***

**Seham S. El-Hawary^1+^, Rabab Mohammed^2+^, Ahmed F. Tawfike^3, 4+^, Sameh Fekry AbouZid^2^, Marwa A. Taher^5^, Usama Ramadan Abdelmohsen^6,7 ⃰^_,_ Elham Amin^8, 9 ⃰^**

*^1^Department of Pharmacognosy, Faculty of Pharmacy, Cairo University, Cairo, Egypt.*

*^2^Department of Pharmacognosy, Faculty of Pharmacy, Beni-Suef University, Beni-Suef 62514, Egypt.*

*^3^ Department of Pharmacognosy, Faculty of Pharmacy, Helwan University, Cairo 11795, Egypt.*

*^4^ Department of Computational and Analytical Science, Molecular Discovery Group, Rothamsted Research, Harpenden AL5 2JQ, England, UK.*

*^5^ Department of Pharmacognosy, Faculty of Pharmacy, Nahda University, Beni-suef, Egypt.*

*^6^Department of Pharmacognosy, Faculty of Pharmacy, Minia University.*

*^7^ Department of Pharmacognosy, Faculty of Pharmacy, Deraya University, Universities Zone, New Minia City, Minia, Egypt.*

*^8^ Department of Pharmacognosy, Faculty of Pharmacy, Nahda University, Beni-suef, Egypt.*

*^9^ Department of Medicinal Chemistry and Pharmacognosy, College of Pharmacy, Qassim University, Buraidah 52471, Saudi Arabia.*

* Corresponding authors

E-mail: [elham_bns@yahoo.com](mailto:elham_bns@yahoo.com), usama.ramadan@mu.edu.eg

+ These authors contributed equally to this work

**Results and discussion**

**Cytotoxic activity of plant extracts**

**Table S1.** IC_50_ (µg/ml) of total ethanolic extracts of plant samples against three cancer cell lines.

| **Plant samples** | **Code** | **HepG2** | **MCF-7** | **CACO2** |
| --- | --- | --- | --- | --- |
| ***T. guayacan* (Seem.) Hemsl. leaf** | Ta-1 | 13.4 | -- | 12.2 |
| ***T. guayacan* (Seem.) Hemsl. stem** | Ta-2 | -- | 14.8 | 13.5 |
| ***T. pulcherrima* leaf** | Ta-3 | 8.6 | 7.3 | 11.2 |
| ***T. pulcherrima* stem** | Ta-4 | 7.9 | 10.6 | 2.6 |
| ***T. rosea* (Bertol.) DC. leaf** | Ta-5 | 9.4 | -- | 8.3 |
| ***T. rosea* (Bertol.) DC. stem** | Ta-6 | 4.7 | -- | 7.6 |
| ***T. pallida* L. leaf** | Ta-7 | 6.3 | 8.7 | 8.3 |
| ***T. pallida* L. stem** | Ta-8 | 7.2 | 6.3 | 9.8 |
| ***T. argentea* Britton leaf** | Ta-9 | 7.2 | 9.6 | 10.3 |
| ***T. argentea* Britton stem** | Ta-10 | 7.1 | 14.7 | 13.2 |

**LC–HRESIMS.** Forty compounds belonging to different chemical classes, in which, Iridoids and phenylethanoids were the major detected chemical classes (Table S2 and S3, Fig. S1-S2). Ten compounds were iridoids, eight were phenylethanoids, five were furanonaphthoquinone derivatives, five were cyclopenten ester derivatives, three were lignans, three were phenylprpanoids, two were benzoyl sugar derivatives, one was a furanobenzocarboxaldehyde, one was a naphthalene derivative, and one was a triterpene.

Both iridoids (**6** – **9**, **12**, **13**, **16**, **17**, **21**, and **31**) and phenylethanoids (**26**–**29**, **34**–**37**), the major chemical classes along with phenylpropanoids (**15**, **38**–**39**) and triterpenoids (**32**), were detected in all plant extracts in the present study. Notably, two iridoids (**21** and **31**) were present at highly relative amount in *T. guayacan* (Seem.) Hemsl*.* stem extract. Furanonaphthoquinone compounds (**1**–**5**) were detected in all extracts except *T. pulcherrima* leaf extract and *T. pallida* L. leaf and stem extracts, supporting notion that these two species are chemically similar, as shown by principal component analysis (PCA) score plotting in the metabolomic analysis. Additionally, the lignan derivatives avellanedae A (**19**), balanophonin 4'-glucoside (**20**), and 4'''-O-methylglehlinoside A (**33**) were detected at high relative amount in *T. pulcherrima* stem extract, *T. pallida* L. stem extract, and *T. pallida* L. leaf extract, respectively. Meanwhile, the unique occurrence of the naphthalene derivative 2-[2, 3-dihydroxy-3-methylbutanoyl]-4-methoxy-1,3-naphthalenediol (**18**) in *T. guayacan* (Seem.) Hemsl. along with the presence of 6-benzofurancarboxaldehyde (**14**) in only *T. rosea* (Bertol.) DC. stem extract also support the unique chemical profiling of these two species. The cyclopentene derivatives **22** and **24** were detected at high relative amount in *T. pallida* L. stem extract, while derivatives **23** and **25** were detected in *T. guayacan* (Seem.) Hemsl. stem extract and derivative **30** was detected in *T. argentea* Britton stem extract. These results prove that the cyclopentene derivatives (**22**–**24** and **30**), previously isolated from *T. rosea* (Bertol.) DC*.* **^1^**, occur at higher relative amount in other species.

**Table S2.** List of secondary metabolites annotated from the total ethanolic extracts of five *Tabebuia* species**.**

| **No** | **Name** | **Chemical formula** | **MS m/z** | **Mode** | **MW** | **Source** | **Rt (min)** | **References** |
| --- | --- | --- | --- | --- | --- | --- | --- | --- |
| **1** | a) 2-acetyl-6-methoxynaphtho[2,3-b]furan-4,9-dione **or**  b) 2-acetyl-7-methoxynaphtho[2,3-b]furan-4,9-dione  **or**  c) 2-acetyl-8-methoxynaphtho[2,3-b]furan-4,9-dione | C_15_H_10_O_5_ | 271.05995 | [M +H]^+^ | 270.0528 | *T. billbergii* (Bureau & K. Schum.) Standl. , *T. ochracea* ssp. *neochrysanta* and  *T. ochracea* | 15.81 | **[2,3]** |
| **2** | 2-acetyl-8-hydroxy-7-methoxynaphtho [2,3-b]furan-4,9-dione | C_15_H_10_O_6_ | 287.05485 | [M +H]^+^ | 286.0477 | *T. ochracea* and *T. billbergii* (Bureau & K. Schum.) Standl. | 14.45 | **[2,4]** |
| **3** | 8-hydroxy-2-(1ꞌ-hydroxyethyl)-7-methoxynaphtho [2,3-b]furan-4,9-dione | C_15_H_12_O_6_ | 287.05612 | [M - H]^-^ | 288.0633 | *T. incana*  A.H. Gentry | 14.85 | **[5]** |
| **4** | a) 2-acetyl-6,7 dimethoxy naphtho [2,3-b]furan-4,9-dione **or**  b) 2-acetyl-7,8 dimethoxy naphtho [2,3-b]furan-4,9-dione | C_16_H_12_O_6_ | 301.0705 | [M +H]^+^ | 300.0633 | *T. ochracea* | 16.07 | **[2]** |
| **5** | 2-(1ꞌ-hydroxyethyl)-7,8-dimethoxynaphtho[2,3-b]furan-4,9-dione | C_16_H_14_O_6_ | 303.08616 | [M +H]^+^ | 302.0790 | *T. ochracea* | 10.64 | **[2]** |
| **6** | 6-*O*-(4-hydroxybenzoyl) ajugol | C_22_H_28_O_11_ | 469.17019 | [M +H]^+^ | 468.1631 | *T. avellanedae*  Lorentz ex Griseb. | 10.68 | **[6]** |
| **7** | 6-*O*-(4-methoxybenzoyl) ajugol | C_23_H_30_O_11_ | 483.18575 | [M +H]^+^ | 482.1788 | *T. avellanedae*  Lorentz ex Griseb. | 11.71 | **[6]** |
| **8** | 6-*O*-(3,4-dimethoxybenzoyl) ajugol | C_24_H_32_O_12_ | 511.18202 | [M - H]^-^ | 512.1893 | *T. avellanedae*  Lorentz ex Griseb. | 9.30 | **[6]** |
| **9** | 6-*O*-(3,4,5-trimethoxybenzoyl) ajugol | C_25_H_34_O_13_ | 541.19267 | [M - H]^-^ | 542.1999 | *T. impetiginosa*  (Mart. ex DC) | 9.96 | **[7]** |
| **10** | 5′-*O*-3, 4-dihydroxybenzoyl-β-D-apiofuranoside | C_12_H_14_O_8_ | 285.06166 | [M - H]^-^ | 286.0688 | *T. avellanedae*  Lorentz ex Griseb. | 7.23 | **[8]** |
| **11** | 5′-*O*-3, 4-dimethoxybenzoyl-β-D-apiofuranoside | C_14_H_18_O_8_ | 315.10725 | [M +H]^+^ | 314.1001 | *T. avellanedae* Lorentz ex Griseb. | 9.10 | **[8]** |
| **12** | Epiaucubin | C_15_H_22_O_9_ | 345.11917 | [M - H]^-^ | 346.1263 | *Tecoma chrysantha* | 7.11 | **[9]** |
| **13** | 6-*O*-(4-hydroxybenzoyl)-6-epiaucubin | C_22_H_26_O_11_ | 465.14021 | [M - H]^-^ | 466.1475 | *T.impetiginosa* (Mart. ex DC) , *Tecoma chrysantha* and  *T. palmeri* | 10.71 | **[9, 10,11]** |
| **14** | 6-benzofurancarboxaldehyde | C_9_H_6_O_2_ | 147.04402 | [M +H]^+^ | 146.0367 | *T. avellanedae* Lorentz ex Griseb. | 13.08 | **[12]** |
| **15** | 1,2-bis (4-hydroxy-3-methoxyphenyl)-1,3-propanediol 4ꞌ-*O*-β –D-glucopyranoside (Hovetrichoside A) | C_23_H_30_O_11_ | 483.18605 | [M +H]^+^ | 482.1788 | *T. impetiginosa* (Mart. ex DC) | 12.83 | **[13]** |
| **16** | 6-*O*-(4-methoxybenzoyl)-crescentin IV 3-*O*-β-D-glucopyranoside | C_23_H_34_O_11_ | 487.21723 | [M +H]^+^ | 486.2101 | *T. impetiginosa* (Mart. ex DC) | 10.38 | **[10]** |
| **17** | 6-*O*-(3,4-dimethoxybenzoyl)-crescentin IV 3-*O*-β-D-glucopyranoside | C_24_H_36_O_12_ | 517.22662 | [M +H]^+^ | 516.2206 | *T. impetiginosa* (Mart. ex DC) | 9.30 | **[10]** |
| **18** | 2-(2,3-Dihydroxy-3-methylbutanoyl)-4-methoxy-1,3-naphthalenediol | C_16_H_18_O_6_ | 307.11751 | [M +H]^+^ | 306.1103 | *T. heptaphylla* (Vell. Conc.) Toledo | 10.79 | **[14]** |
| **19** | Avellanedae A | C_20_H_20_O_7_ | 373.12802 | [M +H]^+^ | 372.1207 | *T. avellanedae* Lorentz ex Griseb. | 13.54 | **[8]** |
| **20** | Balanophonin 4-*O*-β-D-glucopyranoside | C_26_H_30_O_11_ | 519.18492 | [M +H]^+^ | 518.1788 | *T. impetiginosa* (Mart. ex DC) | 10.37 | **[10]** |
| **21** | 10-*O*-(4-methoxybenzoyl)-impetiginoside A | C_23_H_28_O_12_ | 495.15076 | [M - H]^-^ | 496.1580 | *T. impetiginosa* (Mart. ex DC) | 10.75 | **[10]** |
| **22** | 2-formyl-5-(4'-methoxy-benzoyloxy)-3-methyl-2-cyclopentene-1-acetaldehyde | C_17_H_18_O_5_ | 303.12246 | [M +H]^+^ | 302.1154 | *T. impetiginosa* (Mart. ex DC) and *T. rosea* (Bertol.) DC. | 8.30 | **[1, 15]** |
| **23** | Tabebuialdehyde A | C_17_H_18_O_6_ | 319.11748 | [M +H]^+^ | 318.1103 | *T. rosea* (Bertol.) DC. | 11.08 | **[1]** |
| **24** | 2-formyl-5-(3',4 '-dimethoxybenzoyloxy)-3-methyl-2-cyclopentene-1-acetaldehyde | C_18_H_20_O_6_ | 331.11879 | [M - H]^-^ | 332.1259 | *T. impetiginosa* (Mart. ex DC) and *T. rosea* (Bertol.) DC. | 10.18 | **[1, 15]** |
| **25** | Tabebuialdehyde B | C_18_H_18_O_5_ | 315.12257 | [M +H]^+^ | 314.1154 | *T. rosea* (Bertol.) DC. | 11.59 | **[1]** |
| **26** | 2-(4-hydroxyphenyl)ethy1, 1-*O*-β-D-[5-*O*-(4-hydroxybenzoyl)]-apiofuranosyl-(1-6)-β-D-glucopyranoside | C_26_H_32_O_13_ | 551.17691 | [M - H]^-^ | 552.1842 | *T. impetiginosa* (Mart. ex DC) | 10.83 | **[10]** |
| **27** | 2-(4-hydroxyphenyl)ethyl, 1-*O*-β-D-[5-*O*-(3,4-dimethoxybenzoyl)]-apiofuranosyl-(1-6)-β-D-glucopyranoside | C_28_H_36_O_14_ | 595.20306 | [M - H]^-^ | 596.2105 | *T. impetiginosa* (Mart. ex DC) | 11.25 | **[7]** |
| **28** | 2-(4-hydroxyphenyl)ethyl  1-*O*-β-D-[5-*O*-(3,4,5-trimethoxybenzoyl)]-apiofuranosyl-(1-6)-β-D-glucopyranoside | C_29_H_38_O_15_ | 625.21351 | [M - H]^-^ | 626.2210 | *T. impetiginosa* (Mart. ex DC) | 11.15 | **[7]** |
| **29** | 2-(4-hydroxyphenyl)ethyl  1-*O*-β-D-[5-*O*-(4 methoxybenzoyl)]-apiofuranosyl-(1-6)-β-D-glucopyranoside | C_27_H_34_O_13_ | 565.1926 | [M - H]^-^ | 566.1999 | *T. impetiginosa* (Mart. ex DC) | 10.67 | **[7]** |
| **30** | Tabebuialdehyde C. | C_18_H_18_O_7_ | 345.09807 | [M - H]^-^ | 346.1052 | *T. rosea* (Bertol.) DC. | 12.73 | **[1]** |
| **31** | 6-*O*-(4-methoxybenzoyl)-5,7-  bisdeoxycynanchoside. | C_23_H_30_O_12_ | 497.16639 | [M - H]^-^ | 498.1737 | *T. impetiginosa* (Mart. ex DC) | 10.88 | **[10]** |
| **32** | 3β,6β,21β-trihydroxyolean-12-  ene | C_30_H_50_O_3_ | 459.38298 | [M +H]^+^ | 458.3759 | *T. heptaphylla* (Vell. Conc.) Toledo | 25.02 | **[16]** |
| **33** | 4'''-*O*-Methylglehlinoside A | C_35_H_44_O_14_ | 689.27979 | [M +H]^+^ | 688.2731 | *T. impetiginosa* (Mart. ex DC) | 15.45 | **[7]** |
| **34** | 3,4-dimethoxyphenyl 1-*O*-β-D-[5-O-(4-  methoxybenzoyl)]-apiofuranosyl-(1-6)-β-D-glucopyranoside | C_27_H_34_O_14_ | 581.18747 | [M - H]^-^ | 582.1948 | *T. impetiginosa* (Mart. ex DC) | 10.63 | **[7]** |
| **35** | 3,4,5-trimethoxyphenyl 1-*O*-β-D-[5-*O*-(4-  methoxybenzoyl)]-apiofuranosyl-(1-6)-β-D-glucopyranoside | C_28_H_36_O_15_ | 613.2121 | [M +H]^+^ | 612.2054 | *T. impetiginosa* (Mart. ex DC) | 10.97 | **[7]** |
| **36** | 4-hydroxyphenyl 1-*O*-β-D-[5-*O*-(4-  methoxybenzoyl)]-apiofuranosyl-(1-6)-β-D-glucopyranoside | C_26_H_32_O_14_ | 569.18628 | [M +H]^+^ | 568.1792 | *T. impetiginosa* (Mart. ex DC) | 11.14 | **[7]** |
| **37** | 2,4-dimethoxyphenyl 1-*O*-β-D-apiofuranosyl-(  1-6)-β-D-glucopyranoside | C_19_H_28_O_12_ | 447.15089 | [M - H]^-^ | 448.1580 | *T. impetiginosa* (Mart. ex DC) | 6.75 | **[13]** |
| **38** | 2-methoxy-4-[(1*S*,2*S*)-1,2,3-trihydroxypropyl]phenyl 1-*O*-β-D-[6-*O*-(4-hydroxybenzoyl)]-glucopyranoside. | C_23_H_28_O_12_ | 497.16499 | [M +H]^+^ | 496.1580 | *T. impetiginosa* (Mart. ex DC) | 10.72 | **[13]** |
| **39** | 2-methoxy-4-[(1S,2S)-1,2,3-trihydroxypropyl]  phenyl 1-*O*-β-D-[6-*O*-(4-methoxybenzoyl)]-glucopyranoside. | C_24_H_30_O_12_ | 509.16636 | [M - H]^-^ | 510.1737 | *T. impetiginosa* (Mart. ex DC) | 11.07 | **[10]** |
| **40** | Rutin | C_27_H_30_O_16_ | 609.1459 | [M-H]^-^ | 610,1560 | *T. aurea* | 10.65 | **[17]** |


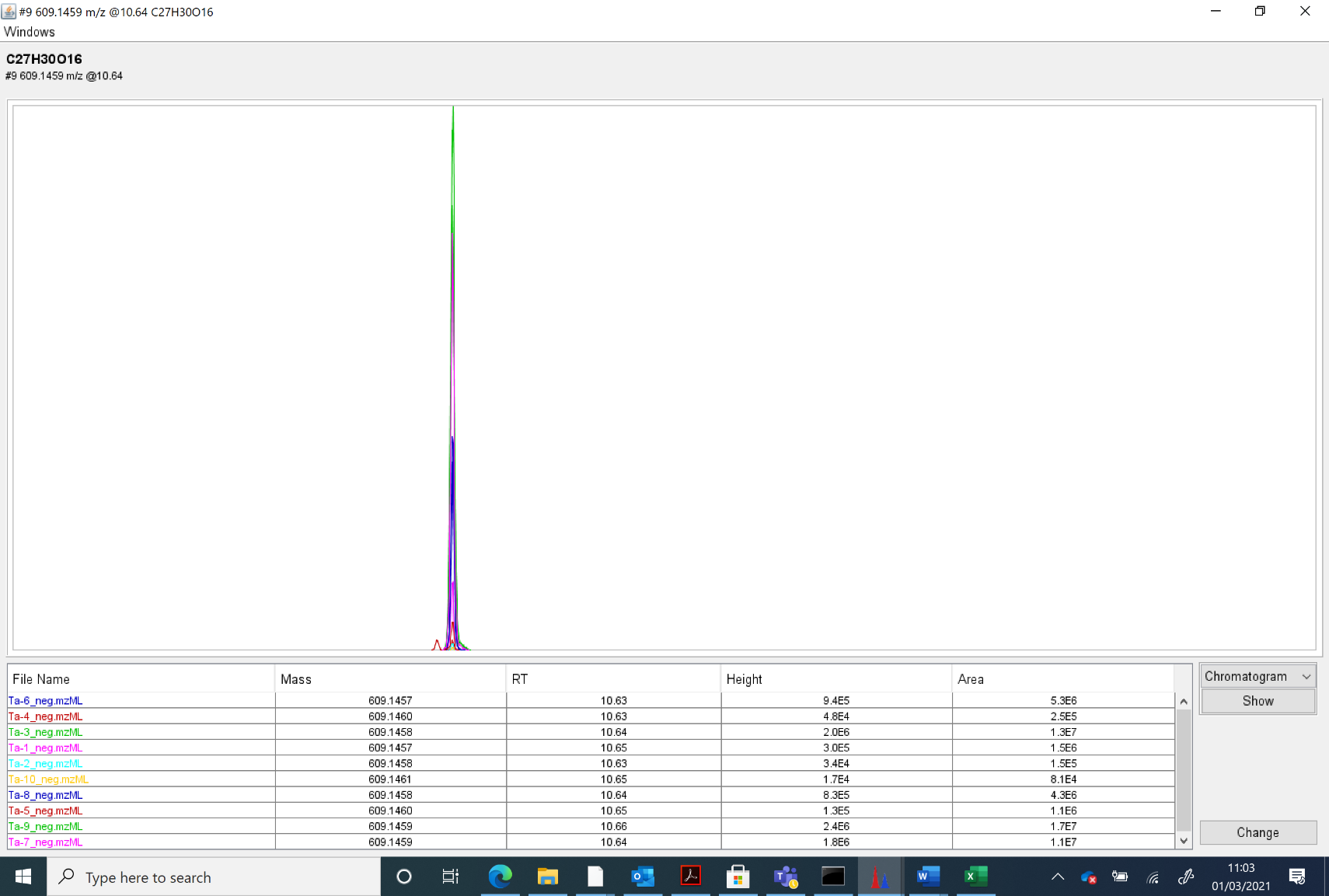


**Figure S1.** The retention time alignment of rutin’s peak in all samples within the acceptable RT tolerance threshold.

**Table S3.** The secondary metabolites annotated among the ten samples of the five *Tabebuia* species under investigation.

| Cpd. NO. | T_1_ | T_2_ | T_3_ | T_4_ | T_5_ | T_6_ | T_7_ | T_8_ | T_9_ | T_10_ |
| --- | --- | --- | --- | --- | --- | --- | --- | --- | --- | --- |
| 1 | + | - | - | **+** | + | + | - | - | + | + |
| 2 | + | + | - | + | **+** | + | - | - | + | + |
| 3 | + | **+** | - | + | + | - | - | - | - | - |
| 4 | + | + | - | **+** | + | + | - | - | + | + |
| 5 | + | + | - | + | **+** | + | - | - | - | + |
| 6 | + | **+** | + | + | + | + | + | + | + | + |
| 7 | + | + | + | + | + | + | **+** | + | + | + |
| 8 | + | + | + | + | + | + | + | + | + | **+** |
| 9 | + | + | + | + | + | + | + | + | **+** | + |
| 10 | + | + | + | + | **+** | + | - | - | - | - |
| 11 | - | + | - | **+** | + | + | - | + | + | - |
| 12 | + | + | + | **+** | + | + | + | + | + | + |
| 13 | + | **+** | + | + | + | + | - | + | + | + |
| 14 | - | - | - | - | - | + | - | - | - | - |
| 15 | + | + | + | + | + | + | **+** | + | + | + |
| 16 | + | + | + | + | + | + | + | + | **+** | + |
| 17 | + | + | + | + | + | **+** | + | + | + | + |
| 18 | + | **+** | - | - | - | - | - | - | - | - |
| 19 | - | - | - | **+** | + | + | - | - | - | + |
| 20 | + | + | + | + | + | + | + | **+** | + | + |
| 21 | + | **+** | + | + | + | + | + | + | + | + |
| 22 | + | + | + | + | + | + | + | **+** | + | + |
| 23 | + | **+** | - | + | + | + | - | + | - | + |
| 24 | - | - | - | - | - | + | - | **+** | - | - |
| 25 | + | **+** | - | + | + | + | + | + | - | + |
| 26 | + | + | + | **+** | + | + | + | + | + | + |
| 27 | + | + | + | + | + | + | + | + | + | **+** |
| 28 | + | **+** | + | + | + | + | + | + | + | + |
| 29 | + | + | + | + | + | + | + | + | + | **+** |
| 30 | - | + | + | + | + | + | + | + | + | **+** |
| 31 | + | **+** | + | + | + | + | + | + | + | + |
| 32 | + | + | + | + | **+** | + | + | + | + | + |
| 33 | - | + | + | - | + | - | **+** | + | - | + |
| 34 | + | + | + | + | + | + | + | **+** | + | + |
| 35 | + | **+** | + | + | + | + | + | + | + | + |
| 36 | + | **+** | + | + | + | + | + | + | + | + |
| 37 | + | + | + | + | + | + | + | + | + | **+** |
| 38 | + | **+** | + | + | + | + | + | + | + | + |
| 39 | + | + | + | + | + | + | + | + | + | **+** |
| 40 | + | + | + | + | + | + | + | + | + | **+** |

T_1_: *T. guayacane* leaf, T_2_: *T. guayacane* stem, T_3_: *T. pulcherrima* leaf, T_4_: *T. pulcherrima* stem, T_5_: *T. rosea* leaf, T_6_: *T.rosea* stem, T_7_: *T. pallida* leaf, T_8_: *T. pallida* stem, T_9_: *T. argentea* leaf and T_10_: *T. argentea* stem; “+” = presence, “-‘’= absence.


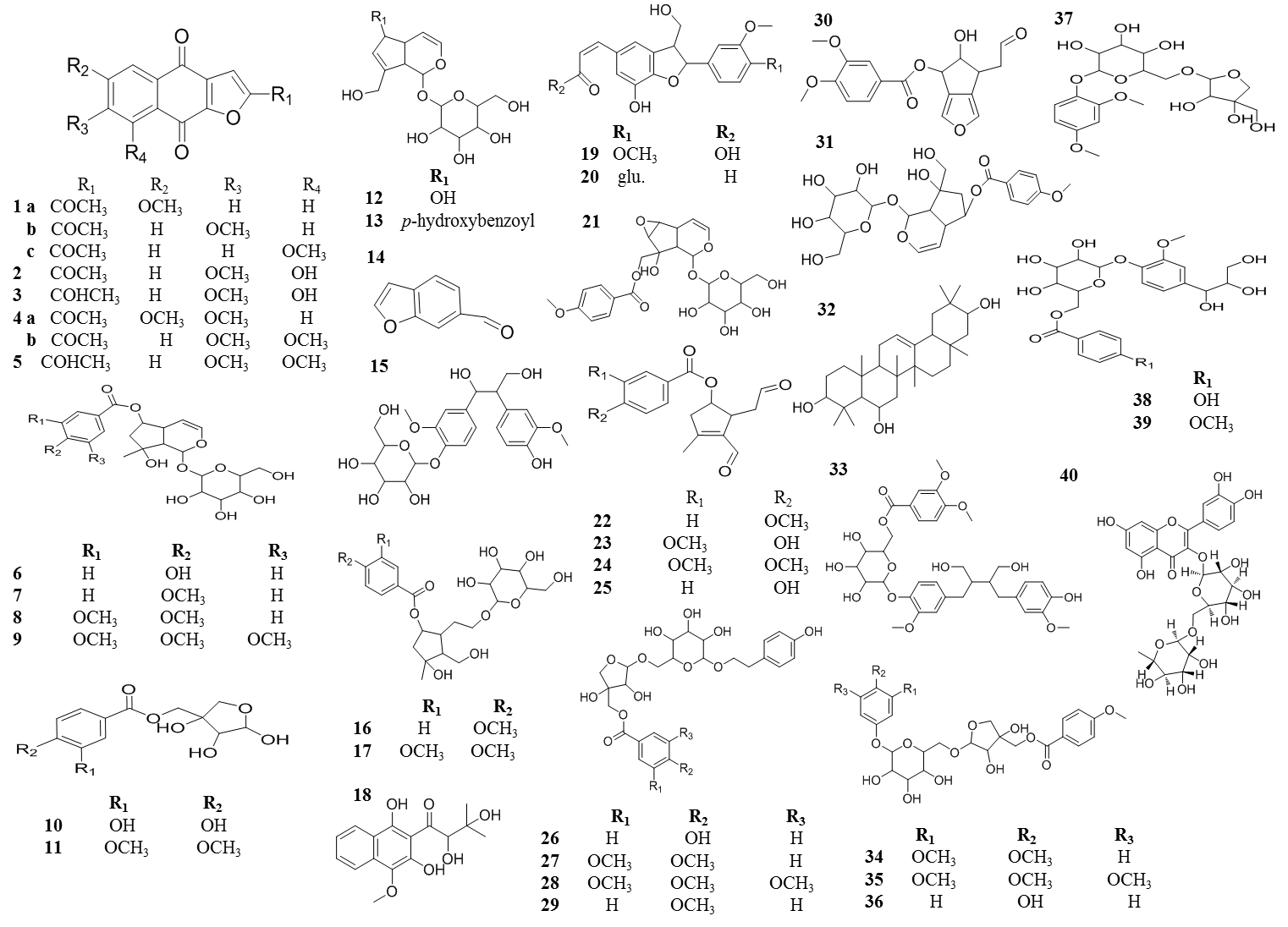
**Figure S2.** Chemical structures of secondary metabolites dereplicated from five *Tabebuia* species

**References**

1. Sichaem, J., Kaennakam, S., Siripong, P. & Tip-Pyang, S. Tabebuialdehydes A–C, cyclopentene dialdehyde derivatives from the roots of *Tabebuia rosea*. *Fitoterapia*, 83(8), 1456-1459 https://pubmed.ncbi.nlm.nih.gov/22926043 (2012).
2. Zani, C. L., De Oliveira, A. B. & De Oliviera, G. G. Furanonaphthoquinones from *Tabebuia ochracea*. *Phytochemistry*, 30(7), 2379-2381 https://www.sciencedirect.com/science/article/abs/pii/0031942291836533 (1991).
3. Díaz, F. & Medina, J. D. Furanonaphthoquinones from *Tabebuia ochracea* ssp. neochrysanta. *J. Nat. Prod.*, 59(4), 423-424 https://pubs.acs.org/doi/abs/10.1021/np9602022 (1996).
4. Gómez-Estrada, H., Gaitán-Ibarra, R., Díaz-Castillo, F., Pérez, H. A. & Medina, J. D. In vitro antimalarial activity of fractions and constituents isolated from *Tabebuia billbergii.* *Rev. cuba. plantas med.*, 17(2), 172-180 https://www.researchgate.net/publication/262651201 (2012).
5. de Oliveira, A. B., Raslan, D. S., de Oliveira, G. G. & Maia, J. G. S. Lignans and naphthoquinones from *Tabebuia incana*. *Phytochemistry*, 34(5), 1409-1412 https://www.sciencedirect.com/science/article/abs/pii/0031942291800394 (1993).
6. ‏ Nakano, K., Maruyama, K., Murakami, K., Takaishi, Y. & Tomimatsu, T. Iridoids from *Tabebuia avellanedae*. *Phytochemistry*, 32(2), 371-373 https://www.sciencedirect.com/science/article/abs/pii/S0031942200949963 (1993).
7. Warashina, T., Nagatani, Y. & Noro, T. Constituents from the bark of *Tabebuia impetiginosa*. *Phytochemistry*, 65(13), 2003-2011 https://www.researchgate.net/publication/7376250 (2004).
8. Suo, M., Isao, H., Kato, H., Takano, F. & Ohta, T. Anti-inflammatory constituents from *Tabebuia avellanedae*. *Fitoterapia*, *83*(8), 1484-1488 https://pubmed.ncbi.nlm.nih.gov/22955001 (2012).
9. Bianco, A., Passacantilli, P., Polidori, G., Nicoletti, M. & de Lima, R. A. New iridoids from *Tecoma heptaphylla*. *Planta Med.*, 45(07), 153-153 https://www.sciencedirect.com/science/article/abs/pii/0040402082801737 (1982).
10. Warashina, T., Nagatani, Y. & Noro, T. Further constituents from the bark of *Tabebuia impetiginosa*. *Phytochemistry*, 66(5), 589-597 https://pubmed.ncbi.nlm.nih.gov/15721952 (2005).
11. Sakhuja, R., Vashist, M., Bhoon, Y. K. & Jain, S. C. Phytochemical investigation of *Tabebuia palmeri*. *Chem. Nat. Compd.* 49(6), 1039-1042 https://link.springer.com/article/10.1007/s10600-014-0818-y (2014).
12. Wagner, H., Kreher, B., Lotter, H., Hamburger, M. O. & Cordell, G. A. Structure Determination of New Isomeric Naphtho [2, 3‐b] furan‐4, 9‐diones from *Tabebuia avellanedae* by the selective‐INEPT technique. *Helv. Chim. Acta*, *72*(4), 659-667 https://onlinelibrary.wiley.com/doi/abs/10.1002/hlca.19890720406 (1989).
13. Warashina, T., Nagatani, Y. & Noro, T. Constituents from the bark of *Tabebuia impetiginosa.* *Chem. Pharm. Bull.*, 54(1), 14-20 https://www.researchgate.net/publication/7376250 (2006).
14. Schmeda-Hirschmann, G. & Papastergiou, F. Naphthoquinone derivatives and lignans from the Paraguayan crude drug “tayï pytá” (*Tabebuia heptaphylla*, Bignoniaceae). *Z. Naturforsch. C*, 58(7-8), 495-501 https://www.degruyter.com/view/journals/znc/58/7-8/article-p495.xml/language=en (2003).
15. Koyama, J., Morita, I., Tagahara, K. & Hirai, K. I. Cyclopentene dialdehydes from *Tabebuia impetiginosa*. *Phytochemistry*, 53(8), 869-872 https://pubmed.ncbi.nlm.nih.gov/10820794 (2000).
16. Garcez, F. R., Garcez, W. S., Mahmoud, T. S., Figueiredo, P. D. O. & Resende, U. M. New constituents from the trunk bark of *Tabebuia heptaphylla*. *Quim. Nova*, 30(8), 1887-1891 https://www.scielo.br/scielo.php?script=sci_abstract&pid=S010040422007000800017&lng=es&nrm=i (2007).
17. Mahmoud, B. K., Hamed, A. N. E., Samy, M. N., Abdelmohsen, U. R., Attia, E. Z., Fawzy, M. A. & Kamel, M. S. Metabolomic profiling and biological investigation of *Tabebuia Aurea* (Silva Manso) leaves, family Bignoniaceae. *Nat. Prod. Res.*, 1-6 https://www.tandfonline.com/doi/full/10.1080/14786419.2019.1698571 (2019).
